# Spectroscopic methods for COVID-19 detection and early diagnosis

**DOI:** 10.1186/s12985-022-01867-2

**Published:** 2022-09-22

**Authors:** Alaa Bedair, Kamal Okasha, Fotouh R. Mansour

**Affiliations:** 1 Department of Analytical Chemistry, Faculty of Pharmacy, University of Sadat City, Sadat City, 32958 Egypt; 2 Internal Medicine and Nephrology Department, Faculty of Medicine, Tanta University, Tanta, Egypt; 3 Department of Pharmaceutical Analytical Chemistry, Faculty of Pharmacy, The Medical Campus of Tanta University, Elgeish Street, Tanta, 31111 Egypt

**Keywords:** COVID-19, Coronavirus, Raman spectroscopy, Infrared spectroscopy, Mass spectrometry, Fluorescence spectroscopy

## Abstract

The coronavirus pandemic is a worldwide hazard that poses a threat to millions of individuals throughout the world. This pandemic is caused by the severe acute respiratory syndrome-coronavirus 2 (SARS-CoV-2), which was initially identified in Wuhan, China's Hubei provincial capital, and has since spread throughout the world. According to the World Health Organization's Weekly Epidemiological Update, there were more than 250 million documented cases of coronavirus infections globally, with five million fatalities. Early detection of coronavirus does not only reduce the spread of the virus, but it also increases the chance of curing the infection. Spectroscopic techniques have been widely used in the early detection and diagnosis of COVID-19 using Raman, Infrared, mass spectrometry and fluorescence spectroscopy. In this review, the reported spectroscopic methods for COVID-19 detection were discussed with emphasis on the practical aspects, limitations and applications.

## Introduction

COVID-19 is a global pandemic caused by the novel severe acute respiratory syndrome coronavirus 2 (SARS-CoV-2). Protection have been the core of pandemic management due to the high transmissibility, the elevated fatality rate (more than 1%) and the lack of viable antiviral treatments [1]. According to the World Health Organization's Weekly Epidemiological Update, there were more than 250 million recorded cases of coronavirus infections globally, with 5 million fatalities [2]. COVID-19 is a pulmonary illness that predominantly affects the endothelium of the blood vessels [3]. In addition, COVID-19 can affect numerous important organs, including the heart, blood vessels, kidneys, abdomen, especially when the patients are not of a vulnerable age. Tremendous efforts have been exerted to slow the COVID-19 spread and to protect all individuals, especially those with chronic diseases [4–9].

COVID-19 identification is regarded as a key step in the eradication and treatment of COVID-19-infected individuals. Furthermore, the quick and thorough testing aids in the tracking and isolation of close contacts of anyone who has tested positive for COVID-19, preventing the virus from spreading. As a result, the test and trace service allow for the management of the reproduction rate, as well as assisting governments in developing plans and implementing required control measures, such as local or nationwide lockdowns, to limit the pandemic and save lives. Several COVID-19 test techniques are currently available in the market [10], which could be divided into two types: viral tests (to identify an infection) and antibody tests (also known as serologic tests). Antigen testing and nucleic acid amplification tests are two forms of viral tests that can be utilized. For the diagnosis of COVID-19 infection, the WHO and the US Centers for Disease Control and Prevention propose real-time reverse transcription polymerase chain reaction (PCR), which involves the detection of viral RNA using nucleic acid amplification tests [11].

Because of its excellent sensitivity and specificity, PCR is the gold standard for detecting COVID-19 infections [12–19]. A PCR test, on the other hand, is a time-consuming procedure that needs a sophisticated laboratory instrument, skilled staff, and sample transportation logistics. Furthermore, the chance of getting false-negative findings from PCR testing might be as high as 29% [20], which became a growing source of worry. Furthermore, delays in receiving PCR findings provide a window in which infectious virus might be shed, making management of onward transmission difficult. Alternative antigen lateral flow assays are faster; however, they have lower sensitivity and specificity, when compared to PCR. Rapid antigen tests, on the other hand, can give important information as point-of-care testing, therefore interest in their performance is increasing, with a particular focus on sensitivity and overall specificity [21]. The antibody-based diagnostic methods are promising, but antibodies may not emerge for weeks or months after the first exposure. As a result, there is still an unmet need for a low-cost, fast diagnostic approach with high sensitivity and specificity that may be used for large-scale testing and monitoring of the COVID-19 infection [22].

Spectroscopic techniques were widely used in virus detection, including Raman [20–22], infrared [23–27], mass spectrophotometry [28–31] and fluorescence spectroscopy [32–34]. In this review, the reported state-of-the-art spectroscopic methods for the COVID-19 detection and early diagnosis were discussed. The different techniques were compared in terms of accuracy, sensitivity and specificity. The requirements, the technical aspects, the limitations and the performance of each technique were discussed.

## Discussion

Spectroscopic methods could be applied to detect the biological changes in COVID-19 infection. The main objective was to develop simple, fast, time saving spectroscopic technique for early detection and diagnosis of coronaviruses. SARS patients' serum samples showed an upregulation of long-chain acylcarnitine (carnitines C18:0 and C18-OH), fructose, myo-inositol phosphate, 1,2,4-trihydroxybenzene, 2,3,4-trihydroxybutyric acid, cysteine and pyroglutamic acid, compared with healthy volunteers. On the other side, methyl esters such as 9,12-octadecadienoic acid and hexadecanoic acid, free carnitine, and arginine were lower in recovered SARS patients, compared with healthy volunteers. In addition, there was an elevation in serum lipids, including phosphatidylglycerol, lysophosphatidylcholines, lysophosphatidylethanolamines and some free fatty acids. Also, infection with COVID-19 resulted in increased blood levels of cysteine, alanine, aspartic acid, succinic acid, and lactic acid, while the levels of ether phosphatidylethanolamines, phosphatidylserines, sphingomyelins, triglycerides and long chain fatty acids decreased in recovered SARS patients [35]. Moreover, angiotensin-converting enzyme II (ACE2); a receptor involved in the virus's entrance mechanism into cells, was highly expressed in oral epithelial cells, according to studies [16, 36]. These changes will be tracked by spectroscopic techniques, as elaborated in in the next sections, to aid in COVID-19 early detection. The most important applications of spectroscopic technique for COVID-19 detection are summarized in Table 1.

**Table 1:** Applications of spectroscopic technique for COVID-19 detection

Sample	Spectroscopic technique	Analyte /Analytes	Sensitivity	Specificity	Accuracy	Ref
Pregnant woman Sera	FTIR	Proteins and lipids	96.30%–100%	91.67%–100%	More than 90%	[55]
Human saliva	ATR-FTIR	Proteins	99.2%	100%	99.6%	[54]
Human Saliva	synchrotron infrared IR and Atomic force microscope AFM-IR	Proteins and lipids	93%	82%	N/R	[50]
Human plasma samples	ATR-FTIR	Proteins	94.1%	69.2%	N/R	[56]
Human saliva samples	ATR-FTIR	Nucleic acids (virus RNA)	95%	89%	90%	[49]
Human nasopharyngeal swab	ATR-FTIR	Virus RNA	97%	98.3	97.8%	[53]
Human nasopharyngeal swab	ATR-FTIR	Proteins and lipids	84% and 87%	66% and 64%	78.4%	[17]
Human serum	ATR − FT-IR	Protein and lipids	87%	98%	N/R	[97]
Human sputum or throat swab	SERS	Virus Antigen	83.3%	92.5%	87.7%	[98]
Nucleic acids samples	SERS	Virus nucleic acids	89.47%	87.5%	N/R	[96]
Human sera	Raman spectroscopy	IgM and IgG	84.0%	95.0%	90.3%	[38]
Human saliva	SERS	Proteins	More than 90%	More than 90%	More than 90%	[16]
Human serum	Raman spectroscopy	Proteins and lipids	87%	100%	93%	[99]
Exhaled breath	PTR-MS	Methylpent-2-enal, 2,4-octadiene 1-chloroheptane, and nonanal	90%	94%	93%	[77]
Exhaled breath	GC–MS	Benzaldehyde, 1-propanol, 3,6-methylundecane, camphene, beta-cubebene, iodobenzene	68%	85%	N/R	[100]
Human plasma	MALDI-TOF–MS	Proteins (amyloid)	93%	92%	92%,	[101]
Nasal swab	MALDI-TOF–MS	Proteins	More than 99%	More than 99%	98.3%	[102]
Nasal swab	MALDI-MS	Proteins (immunoglobulins)	Ranged between 92.7 and 94.5%	Ranged between 90.6 and 92.6%	93.9%	[51]
Human serum	MALDI-TOF–MS	Peptidome Profiling	98%	100%	99%	[88]
Human blood and faecal samples	GC–MS	Sucrose,2-palmitoyl-glycerol, 1,5-anhydroglucitol D-pinitol and oxalate	N/R	N/R	N/R	[103]
Human gargle solution samples	LC/MS	SARS-CoV-2 Proteins	N/R	N/R	N/R	[67]

### COVID-19 virus detection using Raman spectroscopy

Raman spectroscopy is a type of vibrational spectroscopy that could be used to optically examine the molecular alterations in sick tissues. In th-eory, Raman spectroscopy is based on inelastic scattering in which scattered photons have less or more energy than incident photons. The difference in energy corresponds to the vibrational levels in the studied molecules, which gives information about the structural features and alterations in these molecules. Raman spectra are produced by directing a monochromatic laser beam at a sample and plotting the scattered intensity as a function of the energy difference between incoming and scattered photons. The difference in the end and starting vibrational energy levels of the molecules correlates with the loss (or gain) in photon energies. Shifts in wavenumbers (inverse of wavelength) from the incident frequency describe the resulting spectra. The frequency difference between incoming and Raman-scattered light is unique to each molecule and is known as the Raman shift, expressed as cm^−1^ [37].

**Fig. 1:**
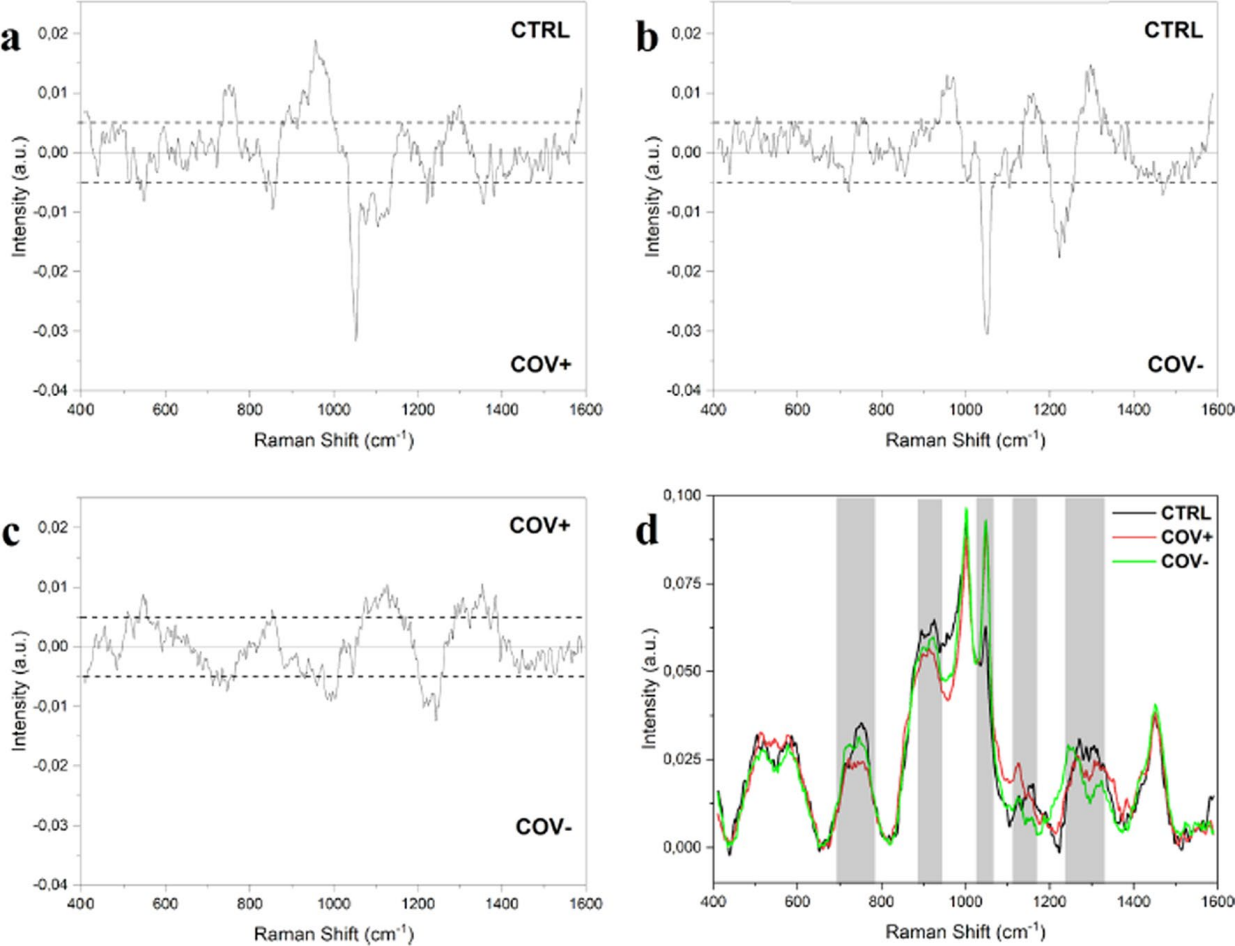
Subtraction Raman spectra of (**a**) the average CTRL signal versus the average signal of COV + , (**b**) the average CTRL signal versus the average COV- spectrum and **c** the average COV + signal versus the average COV − signal. The ± 0.005 ΔI intervals are indicated in the graphs, confirmed by the error propagation from the spectra standard deviation. **d** Overlapped average spectra of CTRL, COV + and COV − with the main different regions highlighted. (With permission from [16])

Raman spectroscopy is a nondestructive analytical method that offers information about the molecular composition of the materials analyzed with only a little quantity of sample and allows diagnosis in a matter of minutes with the right statistical and computational tools [38]. Raman spectroscopy may be used to identify biomolecular characteristics in biological materials, such as lipids, proteins, nucleic acids, and amino acids [39]. Raman spectroscopy, which is based on the principle of inelastic light scattering [40], has been used to identify HIV, dengue fever, and a variety of biomarkers, and could possibly overcome practical issues with mass testing as a point-of-care device [41–43]. For the analysis of biological samples, vibrational spectroscopic techniques such as Raman spectroscopy offer several advantages over biochemical methods, including high speed, the easiness of sample preparation and small sample size required. As mentioned previously, increased blood levels of cysteine, alanine, aspartic acid, succinic acid, and lactic acid were seen after infection with COVID-19 [35], besides angiotensin-converting enzyme II (ACE2), which was significantly expressed in oral epithelial cells of patients [36]. Raman spectroscopy could possibly detect ACE2, by tracking the changes in intensity, width or shifts of Raman peaks [39]. Carlomagno et al. [16] used surface enhanced Raman spectroscopy (SERS) as a novel method for detecting current (COV +) and previous SARS-CoV-2 infections (COV-) in saliva. As indicated in Fig. 1*,* the subtraction spectra were dominated by peaks at 1048 and 1126 cm^−1^, with significant differences between the healthy group and the COV + and COV-, respectively. The strong signals in these two locations were usually linked with an environment high in aromatic amino acids, especially tryptophan and phenylalanine. The same two peaks (1048 cm^−1^ and 1126 cm^−1^) have also been discovered as distinctive signals from coronaviruses, indicating that they are involved in the viral protein structure or interactions with physiologically produced molecules.

**Fig. 2:**
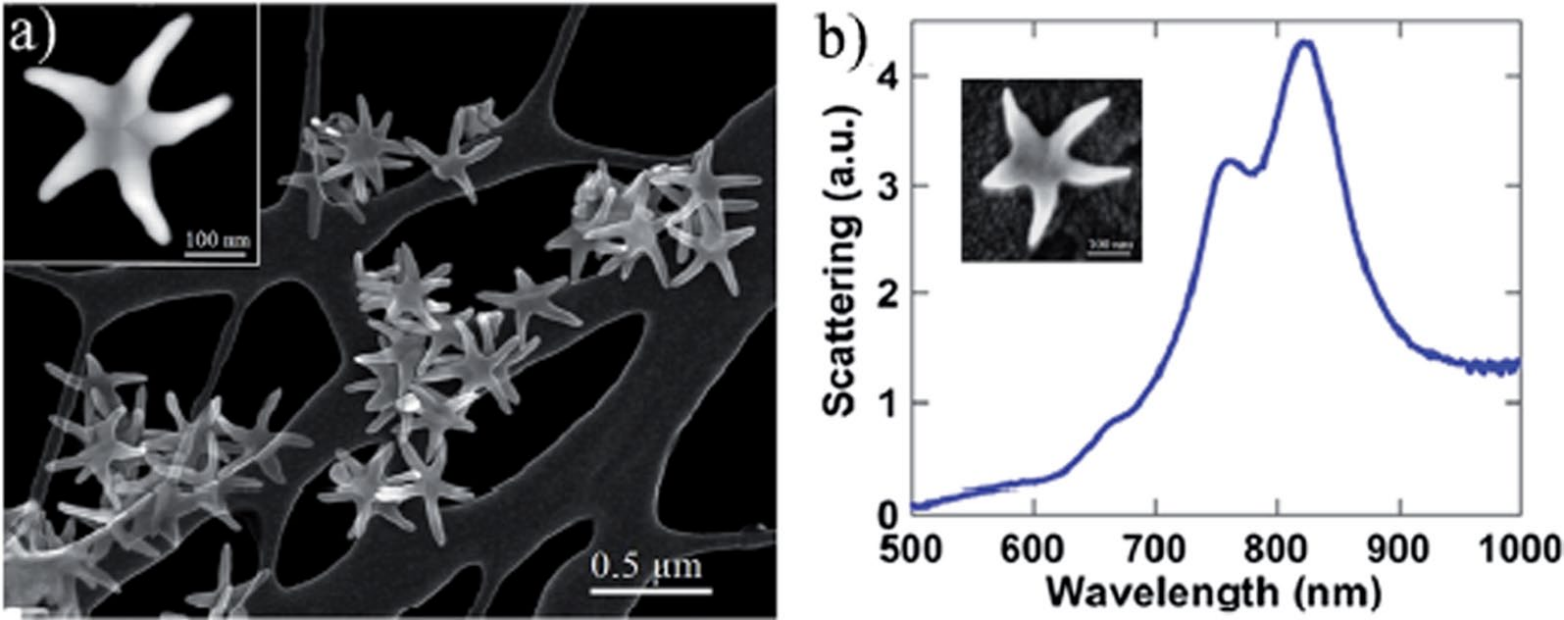
**a** SEM image of the Au–Cu nano, stars used as SERS substrate. The image shows very sharp peaks which act as plasmonic antennas. **b** Optical absorption spectra of the nano, stars revealed a strong peak in the near IR. (With permission from [44])

SERS could be utilized for more than only virus detection, because in it could aid in examining protein structure and track in situ changes caused by various environments. Sanchez et al. [44] used SERS generated by gold nanostars nanoparticles and molybdenum disulfide (MoS_2_) thin films for virus detection (Fig. 2). Furthermore, the major proteins S and N spectra were well-defined. Using a gold nano star, in this case resulted in increasing the signal of SERS. Goulart et al. [38] indicated that Raman spectroscopy could identify COVID-19 in human serum through the biochemical changes associated with the presence of SARS-Cov-2, including an increase in lipids, nitrogen compounds (urea and amines/amides), and nucleic acids, as well as a reduction in proteins and amino acids (tryptophan). These reports indicated that SERS could be a non-invasive, label-free method for detecting COVID-19 infection.

### COVID-19 virus detection using infrared spectroscopy

Infrared spectroscopy is a commonly used analytical method for identifying chemical functional groups in a variety of materials. When infrared light interacts with molecules' intrinsic vibrational modes, it creates a spectrum that represents the sample's chemical fingerprint. Infrared (IR) radiation is the electromagnetic spectrum area between visible (VIS) and microwave wavelengths. Two types of IR radiations are involved in diagnostics; near IR (NIR) and mid IR (MIR). In NIR spectroscopy, molecules are excited at wavelengths ranging from 750 to 2500 nm, corresponding to wavenumbers ranging from 4000 to 13,000 cm^−1^. Solid, liquid, or gaseous samples can absorb certain wavelengths of incoming infrared light, resulting in a fingerprint or a spectrum. Stretching, deformation, and scissor vibrations are performed by molecules with C–H, C–O, C = O, N–H, and O–H functional groups in this spectral area. In contrast to the MIR area (4000–400 cm^−1^), where only fundamental vibrations ("signatures") could be noticed, overtones and combinations are observed in the NIR range, which contains a greater amount of information than MIR. As a result, the spectrum is frequently cluttered with overlapping peaks. Despite the fact that NIR intensities are 10–1000 times lower than MIR intensities, extremely sensitive spectrometers could be developed in a variety of ways, including the use of efficient detectors. The light collected by the detector carries compositional information that a computer may use to report several results almost instantly. IR spectroscopy could allow simultaneous, non-destructive qualitative and quantitative examination of the key components in a wide range of organic compounds [45].

**Fig. 3:**
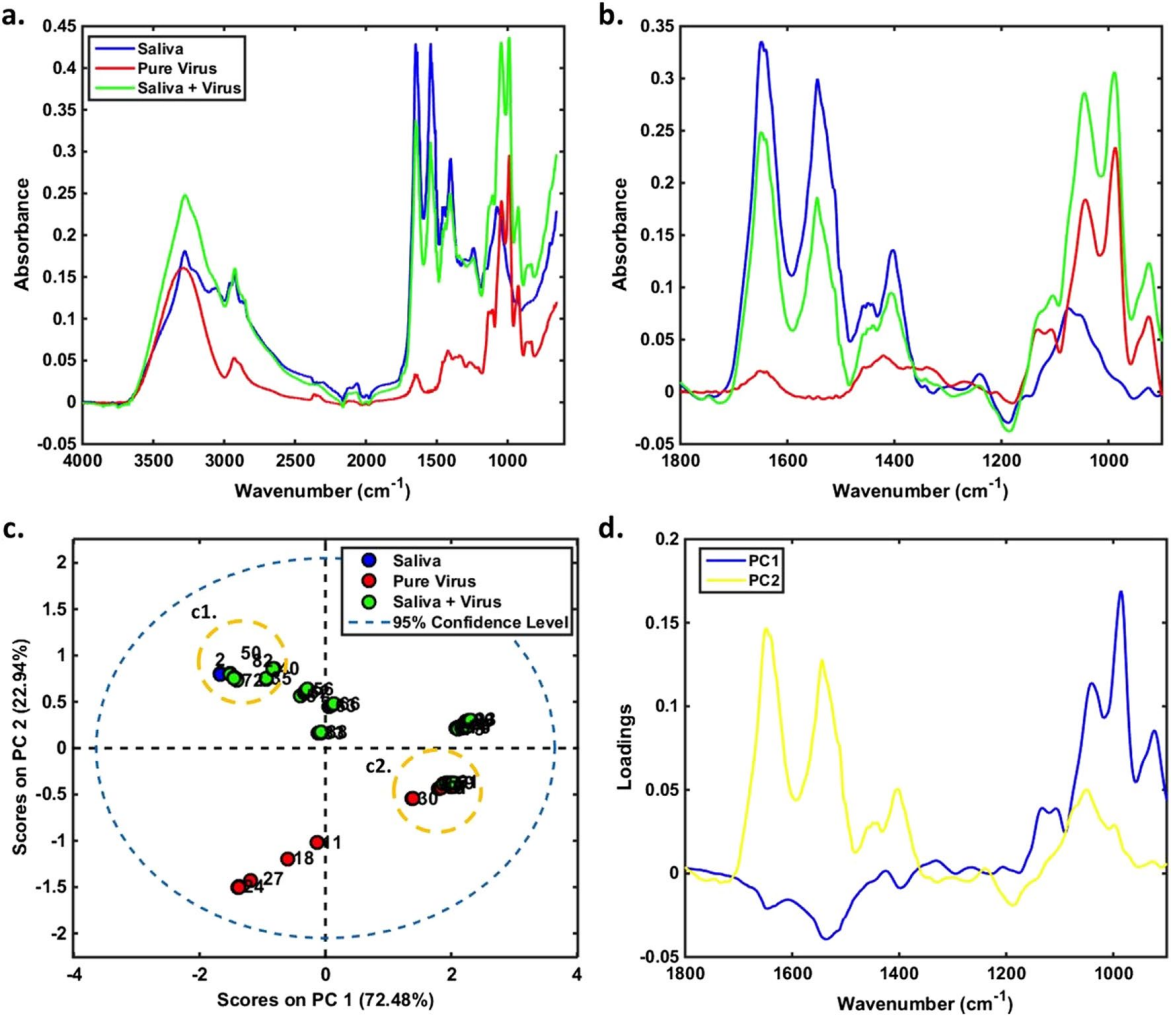
**a** Average raw spectra and **b** pre-processed spectra for saliva (*n* = 2), pure COVID-19 virus in different concentrations (*n* = 28, 1 × 10^5^–98 copies/mL), and saliva + virus in different concentrations (*n* = 63, 1 × 10^5^–24 copies/mL). **c** PCA scores and **d** PCA loadings on PC1 *vs*. PC2 for the pre-processed data. Inset c1 and c2 show both low concentration (≤ 781 copies/mL) and high concentration (≥ 1.25 × 10.^4^ copies/mL) of mixed saliva/virus. (With permission from [49])

In the field of biological diagnostics, the development of portable attenuated total reflection Fourier transform infrared (ATR-FTIR) spectrometers has been prospering over years [46]. ATR-FTIR has recently been used to diagnose Plasmodium species in blood [47] and viruses such as hepatitis B and C in serum [48]. ATR-FTIR relies on identifying both the pathogen's molecular phenotype and the host's immunological response. The chemical differences between infected and uninfected samples could subsequently be used to predict diagnostic outcomes using advanced machine learning algorithms. Barauna et al. [49] used ATR-FTIR to examine saliva obtained from COVID-19 diagnosed individuals. The swab was put on an ATR spectrometer and the spectra of both the saliva and the swab were recorded. Figure 3 demonstrates the examination of swabs spiked with saliva, with or without irradiated COVID-19 virus particles. The bands responsible for increasing concentrations of viral material (nucleic acid bands) are shown on principal component 1 (PC1), whereas the bands responsible for differentiation between saliva and virus are shown on PC2. It is worth mentioning that both the bands of amide I and II bands were observed only in saliva, not in virus.

Saliva contains numerous additional COVID-19 indicators, including ACE2, adenosine deaminase, immunoglobulin G, immunoglobulin M, RNA, and secretory immunoglobulin A, in addition to SARS-CoV-2 virions [50]. Wood et al. [51] investigated these biomarker in saliva for COVID-19 detection. Mir et al. [52] indicated that ATR–FTIR analysis was cost-effective since it took less time and required no reagent. Significant vibrational modes of biological materials generally fell within the 1800–900 cm^–1^ band, which was referred to as the "bio fingerprint" zone.

**Fig. 4:**
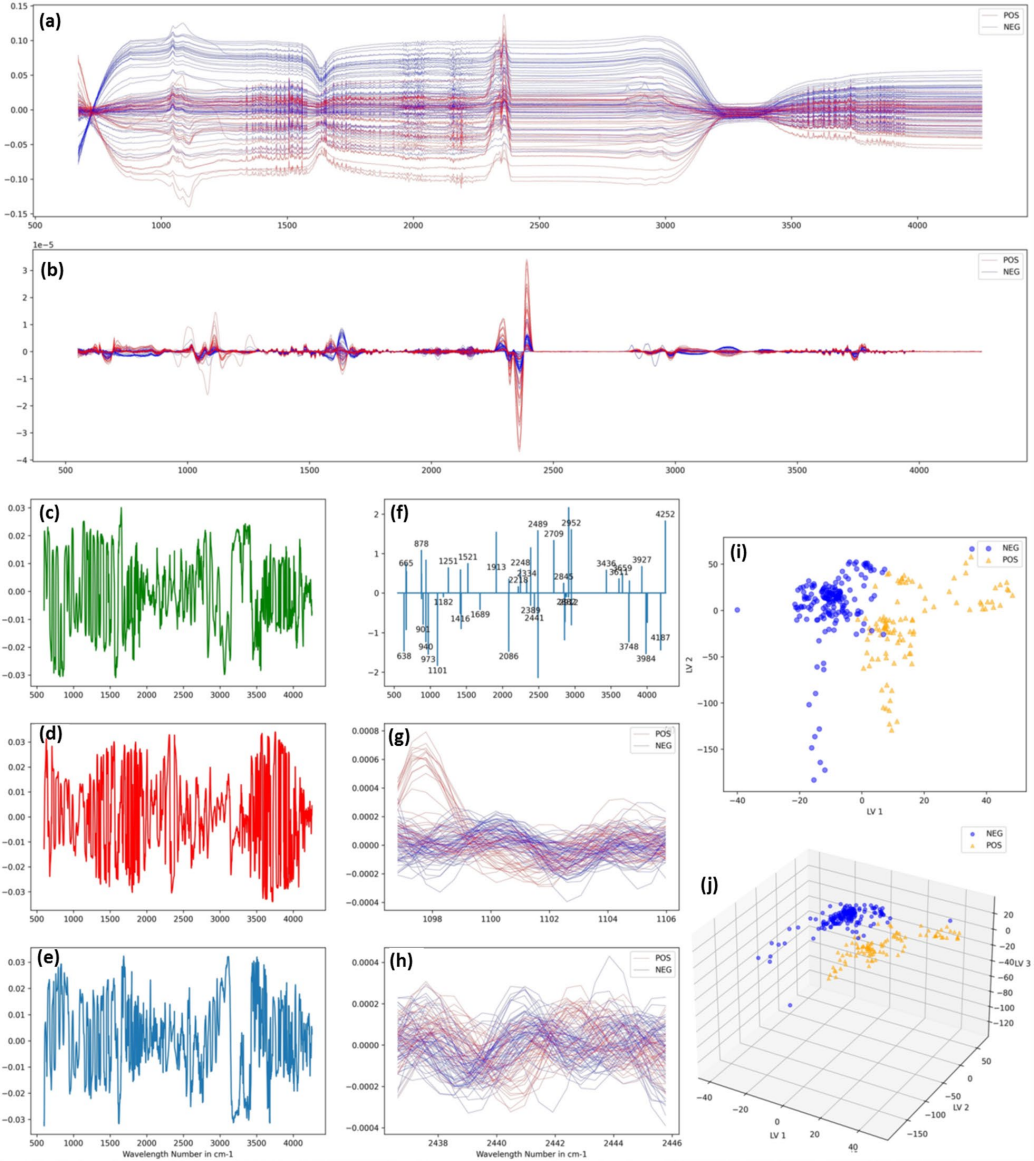
Detection of SARS-CoV-2 with multivariable analysis. **a** Raw Spectra **b** Sample of 2nd derivative of Savitzky–Golay smoothened spectra of positive and negative samples. **c**, **d**, **e** First three latent variables of PLS-DA. (f) Coefficients of variables selected by the sparse classification algorithm of second derivatives of raw spectra (**g**, **h**) Zooms on regions indicated by sparse classification. **i** Projection of the 280 spectra used according to the first two latent variables obtained. **j** Projection of the 280 spectra used according to the first three latent variables obtained. (With permission from [53])

Kitane et al. [53] depended on the determination of SARS-CoV-2 by characterization of extracted RNA samples. Figure 4 indicated that a careful examination of the greatest footprint spectral region of the RNA spectra for positive and negative SARS-CoV-2 RNA samples revealed the existence of three primary visible domains: one at 600–1350 cm^−1^, another at 1500–1700 cm^−1^, and the third at 2300–3900 cm^−1^. The phosphate backbone vibrations (vP-O) were assigned to the first domain, with the 1000–1182 cm^−1^ area resulting from symmetric stretching vibrations of PO_2_ and allocated to the phosphodiester and ribose C–O stretching vibration. The PO_2_ asymmetric stretching vibration of the RNA, which was normally centered at 1251 cm^−1^, could be responsible for the spectral area 1200–1300 cm^−1^. RNA nucleobases may be attributed to the third 1500–1700 cm^−1^ area. In addition, this area coincided with a set of biomarker bands that were commonly associated with Amide I and II vibrations. The stretching vibrations of the OH, NH, and CH groups were aligned in the 2400–3900 cm^−1^ range. Taken together, these results clearly matched the RNA signature, demonstrating the FT-IR/machine learning dual coupling's resilience in viral identification and patient categorization.

**Fig. 5:**
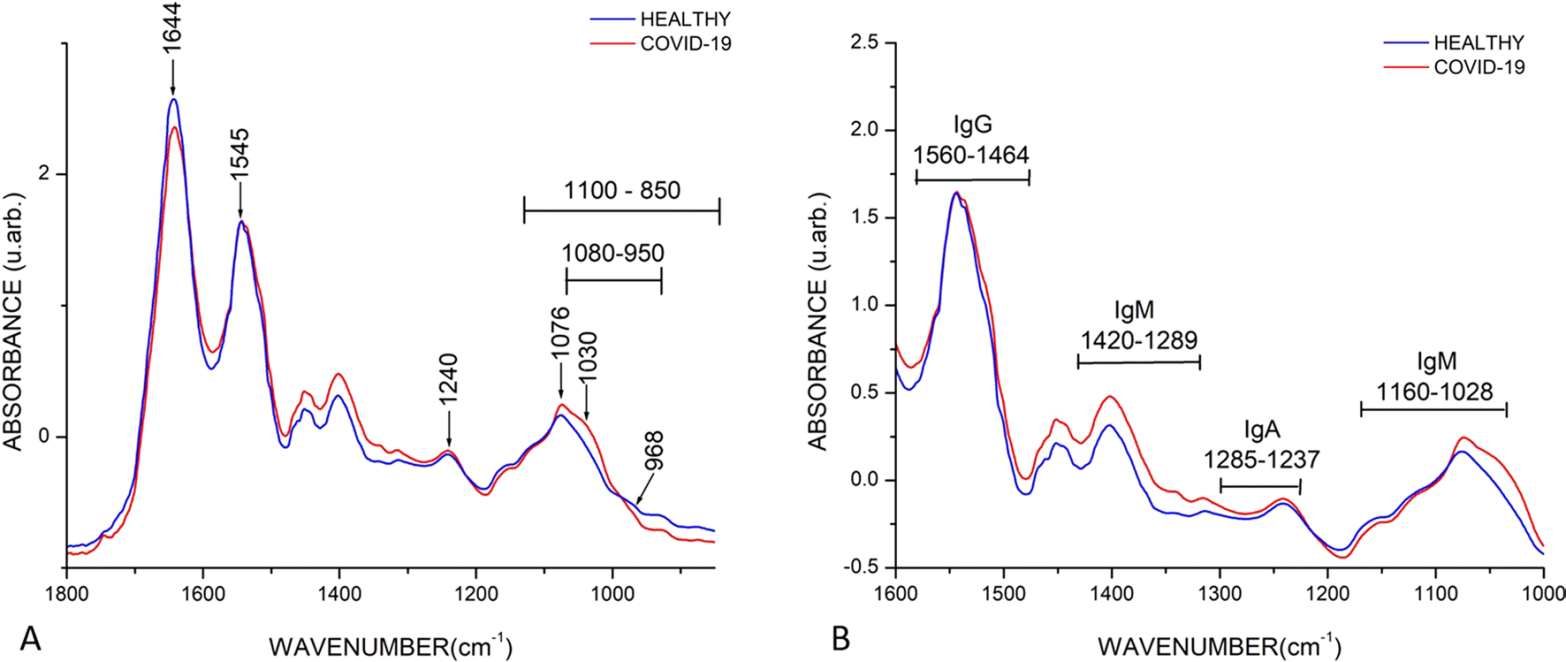
Mean of FTIR spectra of healthy (N = 1209) and COVID-19 (N = 255) groups. **A** Biological fingerprint region, diverse absorption bands related to biological samples are evidenced such as Amide I (1644 cm^−1^), Amide II (1545 cm^−1^), and Amide III (1240 cm^−1^), as well as phosphorylated molecules (1240 cm^−1^and 1076 cm^−1^), carbohydrates (1030 cm^−1^), and DNA backbone (968 cm^−1^). Likewise, peaks in ranges: 1100–850 cm^−1^and 1080–950 cm^−1^ attributed to nucleic acids and α-amylase, respectively were observed. Differences in absorbance and displacements between the bands of the groups representing changes in biochemical compositions were evidenced. **B** Immunoglobulins regions, different intervals were detected such as IgG (560–1464 cm^−1^), IgM (1420–1289 cm^−1^ and 1160–1028 cm^−1^), and IgA (1285–1237 cm.^−1^), noticing that the COVID-19 group exhibited a higher absorbance. (With permission from [54])

Cuazitl et al. [54] used ATR-FTIR for the determination of immune responses through three different antibodies. IgA, IgG and IgM were assigned to the regions 1285–1237, 1560–1464 and 1420–1289 cm^−1^, respectively as indicated in Fig. 5.

Guleken et al. [55] used FTIR spectroscopy to prove that COVID-19 affected peripheral blood cells, biochemical parameters, and coagulation markers in both second and third trimester pregnant women. Furthermore, COVID-19 lowered albumin levels in both third trimester and severe second trimester women. Banerjee et al. [56] invested ATR-FTIR for COVID-19 severity classification. The study indicated that ATR-FTIR was a promising method for COVID-19 monitoring in real time. Clinical laboratory professionals may readily carry out the sample preparation and spectrum collection procedures established here due to their simplicity. The high cost of the instruments could be the main obstacle against the widespread of Raman spectroscopy in COVID-19 diagnosis.

### COVID-19 virus detection using fluorescence spectroscopy

Luminescence is the emission of light from any substance that occurs when it is stimulated by light. Luminescence is classified into two types: fluorescence and phosphorescence, based on the nature of the excited state. The electron in the excited orbital is coupled (by opposing spin) to the second electron in the ground-state orbital in excited singlet states. As a result, return to the ground state is spin permitted and occurs swiftly via photon emission. Fluorescence emission rates are approximately 108 s^−1^, resulting in a typical fluorescence lifetime of around 10 ns (10 × 10^−9^ s). A fluorophore's lifespan is the average duration between excitation and return to the ground state. Because fluorescence has a short timeframe, measuring time-resolved emission needs complex optics and electronics. Despite the extra complexity, time-resolved fluorescence is extensively employed because the data contain more information than stationary or steady-state observations. Furthermore, technological advancements have simplified time-resolved measurements, even when utilizing microscopes [57]. The development of fluorescence-based optical technology has been widely used in biomedical for illness detection and diagnosis [15].When given a light source with a wavelength of 200–800 nm, the samples will glow and provide a response in the form of a conventional fluorescence spectrum with excitation and emission peaks [58]. Disease diagnosis was traditionally done by identifying the primary cause of disease, such as germs, anomalies in certain bodily cells, or viruses. When a virus has multiplied itself in vivo, detection of illnesses caused by viruses is possible. Virus genetic information is dependent on the genetic information of virus-infected organisms. For each of these viruses, genetic information can be a unique marker that appears as a fluorescence emission[33]. To identify the virus, a 450 nm laser diode was employed as a light source. Using a labelling agent, the virus's specific fluorescence emission may be boosted. Rong et al. created a smartphone-based fluorescent lateral flow immunoassay (LFIA) technology for extremely sensitive Zika virus nonstructural protein 1 point-of-care detection [59]. In this investigation, ZIKV NS1 was detected quantitatively at the point of care in 20 min. ZIKV NS1 had detection limits of 0.045 and 0.15 ng/mL in buffer and serum, respectively. Fluorescence technology was used for more than just the initial detection and screening. It can, however, be utilized to describe coronavirus enzymatic activity. In 2005, Chen et al. employed fluorescence resonance energy transfer to study the enzymatic activity of the coronavirus (3CLpro) 3C-like protease (3CLpro) and its four sites directed mutations in the severe acute respiratory syndrome (SARS) coronavirus (CoV) [60]. Kong et al. (2015) employed a bimolecular fluorescence complementation (BiFC) test to discover SARS-CoV accessory protein interactions. The universal application of the BiFC system for the evaluation of protein–protein interactions of SARS-CoV with direct visualization was demonstrated in this work. Fluorescence technology can be used in conjunction with other approaches to identify coronaviruses. To detect SARS-CoV, Huang et al. developed a fiber-optic biosensor with a localized surface plasmon coupled fluorescence (LSPCF) [61]. Fluorescence technology can also be employed in the diagnosis of COVID-19 infections. In 2020, Diao et al. developed an immunochromatographic fluorescent method for identifying SARS-CoV-2 nucleocapsid protein [62].

### COVID-19 virus detection using mass spectroscopy

Mass spectroscopy is an analytical method used to identify biomolecules or proteins in biological materials, as well as to study protein–protein interactions. The fundamental idea is that a substance or molecule is fragmented into charged species, which are then accelerated, deflected, and eventually focused on a detector based on their mass and charge ratio. Ion deflection is determined by charge, mass, and velocity, while ion separation is monitored by the mass to charge (m/z) ratio, and the signal intensity is proportional to the ion abundance [63]. Mass spectrometry (MS) is a useful analytical method for examining genomes, proteomics, metabolomics, and microbiomics of human disorders [64, 65] due to its inherent sensitivity, specificity, and speed. These advantages makes MS-based technologies a valuable analytical tool to detect COVID-19 [66]. On COVID-19-related human fluids, significant MS-based metabolomic and proteomic research have been completed [67–74] using different ionization techniques such as matrix-assisted laser desorption/ionization (MALDI), ambient ionization, desorption electrospray ionization (DESI) [75], paper spray [76]) and direct ionization [77].

In clinical and pathologic research on COVID-19, evaluating human exhaled breath aerosol (EBA) profiles is beneficial due to COVID-19's respiratory characteristics [78, 79]. Volatile organic compounds, droplets that can dissolve a variety of non-volatile metabolites, salts, proteins, and microorganisms such as bacterial and viral particles were all present in EBA, which could be a substantial source of coronavirus infection [80]. MS Spectrometry-based approaches of human breath samples have numerous benefits over existing COVID-19 diagnostic procedures being noninvasive, easy to operate, with a strong analytical performance. Grassin-Delyle et al. used PTR-MS to detect volatile organic compounds (VOCS) in breath including 2,4-octadiene 1-chloroheptane, methylpent-2-enal and nonanal. This study found that direct MS analysis of breath VOCs from COVID-19 patients had a 93% accuracy, which could lead to the development of the novel online approaches for large-scale COVID-19 screening [77]. For COVID-19 diagnosis, GC–MS analysis of breath VOC and blood metabolites has been recommended [81, 82]. LC–MS has been a widely used technique for determination of exhaled breath condensate (EBC). This EBC contained a range of nonvolatile organic chemicals and biological matrices that could be used to provide useful biochemical information regarding respiratory disorders in the future. LC–MS has therefore been proposed for the COVID-19 diagnosis [82]. Because SARS-CoV-2 can be transmitted by breath droplets, direct detection of SARS-CoV-2 from breath samples is critical [83]. Because of its excellent precision, mass range, and good analytical performance, MALDI-MS is an efficient analytical instrument for detecting microorganisms such as bacteria, fungi, and viruses [84–87]. So, MALDI-MS is expected to a powerful diagnostic tool using breath sample. Nachtigall et al. used MALDI-MS for COVID-19 detection by using a nasal swab. The results showed that MALDI-MS is an accurate, sensitive, cheap and simple in comparison with RT-PCR [51]. Yan et al. used Matrix assisted laser desorption/ionization time-of-flight mass spectrometry (MALDI-TOF MS) for fast detection of COVID-19 depending on peptidome profiling of serum. The results showed that COVID-19 may be detected quickly and accurately using MALDI-TOF-based serum profiling. It offers a lot of promise for screening, regular surveillance, and diagnostics in big populations, which is crucial for pandemic control [88].

## Perspectives

**Fig. 6:**
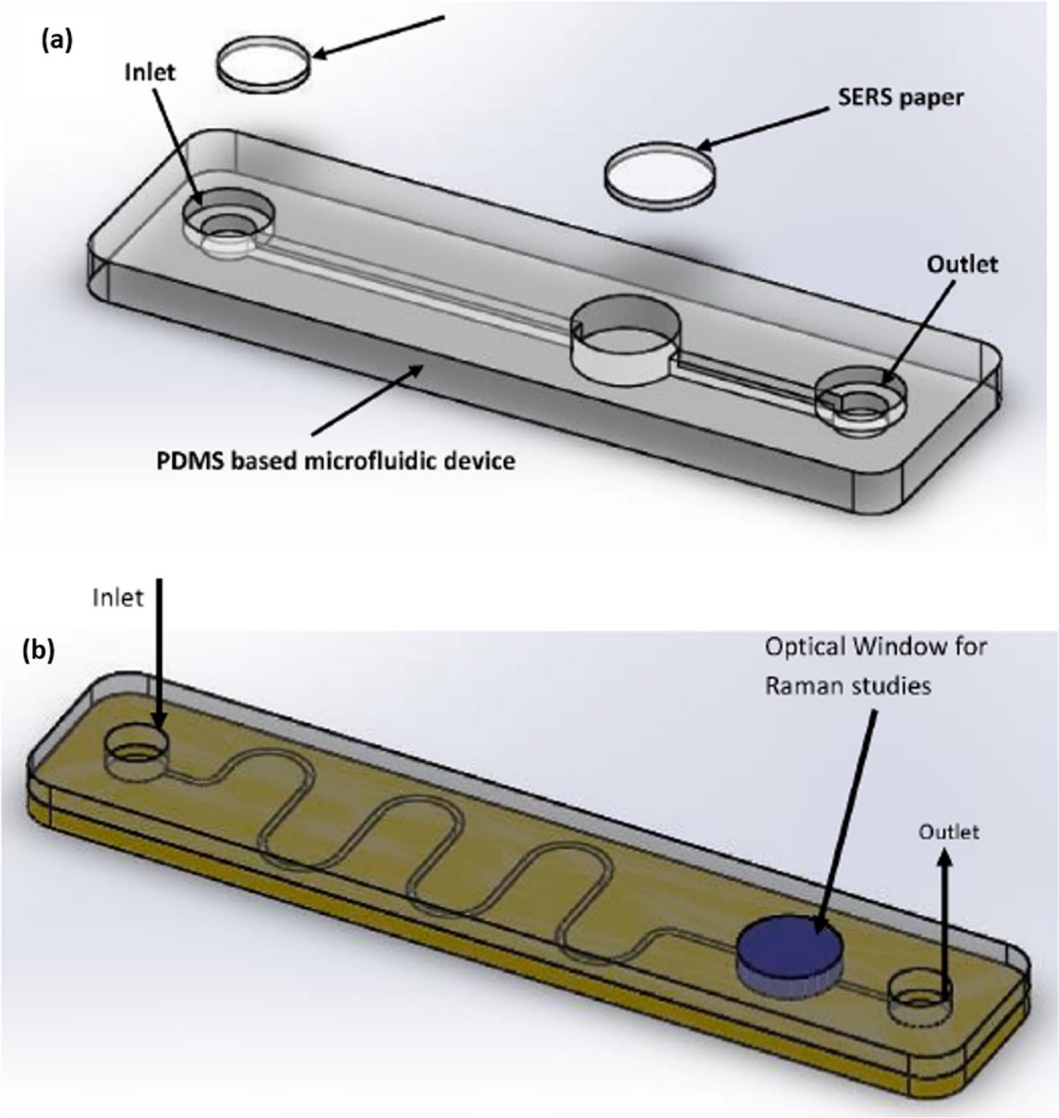
**a** Proposed microfluidic platform for virus detection. **b** Proposed VACNTs functionalized microfluidic platform. (With permission from [14])

Microfluidics has found applications in a variety of sectors, including medication delivery, clinical diagnostics, and chemical analysis [89]. A microfluidic platform is made up of micro-scale fluid handling compartments including channels, valves, reservoirs, membranes, and so on that allow for integrated biochemical examination in a consistent and straightforward manner [90, 91]. Jadhav et al. used SERS in conjunction with microfluidic devices with built-in microchannels as indicated in Fig. 6. The Raman signal could be enhanced by the Ag/Au nanoparticles in the detecting well. Machine learning techniques could be used to analyze the Raman signal, which would be trained on the signals of known viral molecules. The spectrum of the test sample would be compared to a previously recorded SARS-CoV-2 standard reference spectrum. Viruses are trapped using vertically aligned Au/Ag coated carbon nanotubes (Au/Ag-VACNTs), which not only help to separate viruses from samples, but they also help to concentrate them (on repeated applications). This localized rise in titre aids in the correct identification and increases the assay's sensitivity. This assay does not need the tagging of virus particles or the preparation of materials before they are introduced into the instrument. As a result of the short time, the microfluidic-based SERS has the potential to become a robust, reliable, highly sensitive, and extremely quick detection technique of choice throughout the world. Furthermore, the excellent performance of this tool will make it easier to build and manufacture portable systems to combat future viral outbreaks with a significant reduction in reaction time [14].

SARS-CoV-2 diagnosis can be derived from mRNA or microRNA (miRNA) detection approaches, using a programmable nanoparticle network made up of DNA-bridged gold nanoparticles and quantum dots to boost signal gain [92, 93]. Quantum dots made of carbon and graphene have unique electrical and fluorescent characteristics that may be attached to RNA or DNA substrates to detect viral RNA. Carbon dots are more favorable than traditional quantum dots and organic dyes because of their excellent water solubility, photobleaching stability, greater biocompatibility, and low toxicity [94, 95]. Yao et al. [96] depended on single-stranded DNA probes, functionalized with evaporated gold nano-island sheets as surface enhanced infrared absorption (SEIRA) substrates for targeted binding to certain SARS-CoV-2 genomic regions. To find the important distinctive changes between infected and control samples, the infrared absorption spectra were examined using the principal component analysis approach. The SEIRA-based biosensor could detect SARS-CoV-2 quickly, taking less than 5 min to detect 1 million viral nucleic acids without any amplification. The detection capability of 2.98 copies per liter may be performed in 30 min when paired with the recombinase polymerase amplification therapy. This method provides a straightforward and cost-effective method for diagnosing COVID-19, which would be beneficial in monitoring and managing future pandemics in a timely way.

## Conclusion

In the fight against COVID-19, scientists have gone a long way. However, a new viral variety might jeopardize development. Viruses are transferred more easily through human contact and touching surfaces. As a result, quick and accurate viral identification methods are essential in order to limit pandemic breakouts. This review gave an overview of COVID-19 and a comprehensive knowledge of coronavirus detection utilizing spectroscopic tools. These spectroscopic methods are rapid and practical, for determination of COVID-19 in comparison with real time PCR which is the gold standard diagnosis.

## Data Availability

Not applicable.
